# Demonstrating aspects of multiscale modeling by studying the permeation pathway of the human ZnT2 zinc transporter

**DOI:** 10.1371/journal.pcbi.1006503

**Published:** 2018-11-02

**Authors:** Yarden Golan, Raphael Alhadeff, Fabian Glaser, Assaf Ganoth, Arieh Warshel, Yehuda G. Assaraf

**Affiliations:** 1 The Fred Wyszkowski Cancer Research Laboratory, Dept. of Biology, Technion-Israel Institute of Technology, Haifa, Israel; 2 Department of Chemistry, University of Southern California, Los Angeles, CA, United States of America; 3 Lorry I. Lokey Interdisciplinary Center for Life Sciences and Engineering, Technion-Israel Institute of Technology, Haifa, Israel; 4 The Interdisciplinary Center (IDC), Herzliya, Israel; University of Maryland, UNITED STATES

## Abstract

Multiscale modeling provides a very powerful means of studying complex biological systems. An important component of this strategy involves coarse-grained (CG) simplifications of regions of the system, which allow effective exploration of complex systems. Here we studied aspects of CG modeling of the human zinc transporter ZnT2. Zinc is an essential trace element with 10% of the proteins in the human proteome capable of zinc binding. Thus, zinc deficiency or impairment of zinc homeostasis disrupt key cellular functions. Mammalian zinc transport proceeds via two transporter families: ZnT and ZIP; however, little is known about the zinc permeation pathway through these transporters. As a step towards this end, we herein undertook comprehensive computational analyses employing multiscale techniques, focusing on the human zinc transporter ZnT2 and its bacterial homologue, YiiP. Energy calculations revealed a favorable pathway for zinc translocation via alternating access. We then identified key residues presumably involved in the passage of zinc ions through ZnT2 and YiiP, and functionally validated their role in zinc transport using site-directed mutagenesis of ZnT2 residues. Finally, we use a CG Monte Carlo simulation approach to sample the transition between the inward-facing and the outward-facing states. We present our structural models of the inward- and outward-facing conformations of ZnT2 as a blueprint prototype of the transporter conformations, including the putative permeation pathway and participating residues. The insights gained from this study may facilitate the delineation of the pathways of other zinc transporters, laying the foundations for the molecular basis underlying ion permeation. This may possibly facilitate the development of therapeutic interventions in pathological states associated with zinc deficiency and other disorders based on loss-of-function mutations in solute carriers.

## Author summary

Herein we employed multiscale modeling and electrostatic energy calculations to delineate, for the first time, a putative zinc permeation pathway, from the cytoplasm into intracellular vesicles (for ZnT2) or to the extracellular milieu (for YiiP), along the membrane-spanning domain of the human zinc transporter ZnT2 and its *E*. *coli* homologue, YiiP. These computational findings were functionally validated using site-directed mutagenesis of ZnT2 residues predicted to reside along the putative zinc permeation pathway and zinc transport assay. Our results shed light on the transport mechanisms of ZnT2 and YiiP and pave the way towards the elucidation of the zinc translocation mechanism in other ZnT family members. Furthermore, these findings could also be harnessed to the possible development of therapeutic interventions in zinc-associated pathologies.

## Introduction

Multiscale computer simulations provide a general philosophy that allows one to explore complex systems while choosing the proper level of details for different regions of the system [1]. Thus, such approaches use coarse-grained (CG) treatments where molecular systems are simplified by, for example, treating groups of atoms as a single particle, to decrease the required resources and allow longer or larger simulations. The success of such systems in predicting and explaining biological phenomena has been exemplified by many studies of complex systems (e.g. [1–5]). Studying a system at CG-level resolution is particularly beneficial when the systems are very large, the process investigated takes place over long time-scales (≥ microseconds), or when the system is of low resolution. In the latter case, the advantage of CG modeling is that it does not treat all atoms explicitly, and therefore low-resolution structures (commonly cryo-EM) or models (with the accompanied uncertainties and inaccuracies) are good examples where CG modeling can perform better than full atomistic simulations (e.g. [6]). In fact, in studies of complex systems it is recommended to start by charting the system with CG modeling. Lastly, the advantage of CG is that it results in a smoother landscape and thus results in faster convergence, which is arguably one of the key requirements in computational biology simulations.

In the current study we demonstrate the use of several aspects of multiscale modeling by studying the physiologically important human zinc transporter ZnT2 and investigating its permeation pathway. Zinc is the second most abundant trace element in the human body and it is estimated that over 10% of the proteins in the human proteome are capable of zinc binding [7]. Zinc is crucially important for numerous physiological processes including metabolism of nucleic acids, regulation of gene expression, signal transduction, cell division, immune- and nervous-system function, wound healing and apoptosis [8]. In humans, intracellular zinc homeostasis is tightly regulated via the transport functions of two transporter families containing 24 different transmembrane carriers, ZIP1-14 and ZnT1-10 [9]. Moreover, various metalloproteins bind free zinc, hence buffering cytoplasmic zinc levels [10].

In the past decade, the human zinc transporter 2 (ZnT2/SLC30A2) was found to be the predominant transporter mediating the translocation of zinc into breast milk in lactating mammary epithelial cells [11], involved in clinical cases of transient neonatal zinc deficiency (TNZD) [11–19]. Specifically, mothers harboring loss of function mutations in ZnT2 produce breast milk containing very low zinc levels; consequently, their exclusively breastfed infants suffer from severe zinc deficiency. These TNZD infants present with dermatitis, diarrhea, alopecia, loss of appetite, and consequently display growth and developmental delays [9]. Importantly, the unaffected healthy ZnT2 allele in TNZD was recently shown to be insufficient to provide the necessary high levels of zinc in breast milk, which are strictly required for proper infant growth and development [9,16]. Currently, no zinc transporter other than ZnT2 is known to play such a vital role in zinc transport into human breast milk.

Taking into consideration the crucial role of ZnT2 in human health, we undertook the current study to understand the molecular mechanism underlying transmembrane zinc transport through ZnT2. As a step towards this end, we used multiscale computational analyses and structural modeling to delineate the zinc permeation pathway and study the conformational dynamics of the transporter. We then functionally validated our proposed permeation pathway using site-directed mutagenesis and experimental zinc transport assays. To date, there is no high-resolution structure of the clinically significant ZnT2 transporter. However, *E*.*coli*’s YiiP, the closest ZnT2 homologue with a known crystal structure, is assumed to harbor a similar 3D structure [13]. YiiP and ZnT2 share ~20% sequence identity (~28% similarity) along the region aligned (ZnT2 residues 70–372), allowing homology-based 3D model reconstruction of ZnT2. Interestingly, YiiP was recently suggested to be involved in antibiotic resistance in *Pseudomonas Aeruginosa* [20] which enhances the importance of identification of the ion permeation pathway of YiiP as well. Herein, we conducted an array of calculations on the available structures of the bacterial YiiP (X-ray and cryo-EM), and the 3D model of the human ZnT2 both in the inward- and outward-facing conformations. We investigated the zinc permeation pathway with different multiscale strategies, ranging from PDLD/S-LRA binding free energy calculations, CG evaluation of conformational change process and Monte Carlo simulations. It should be noted, with respect to zinc binding, that the large experimental free-energy of solvation for a zinc ion (-467 kcal/mol [21,22]) should be compensated by interactions with the transporter residues, and this is a computationally challenging task which was handled in our case by using explicit ligand particles (see below as well as the Methods section). We complemented our computational work with site-directed mutagenesis, subcellular localization and functional zinc transport assay to delineate the putative zinc permeation pathway, highlighting key residues predicted to facilitate transmembrane zinc ion translocation through ZnT2.

The current study demonstrates the strengths of multiscale modeling and highlights the benefits of combining computational and experimental approaches to address medically important questions. We present the first proposed zinc permeation pathway of a human zinc transporter, bearing important implications for pathophysiological states of zinc deficiency and possible development of proper therapeutic interventions for zinc deficiency-associated disorders.

## Results

### Exploration of the zinc permeation pathway

**Fig 1:**
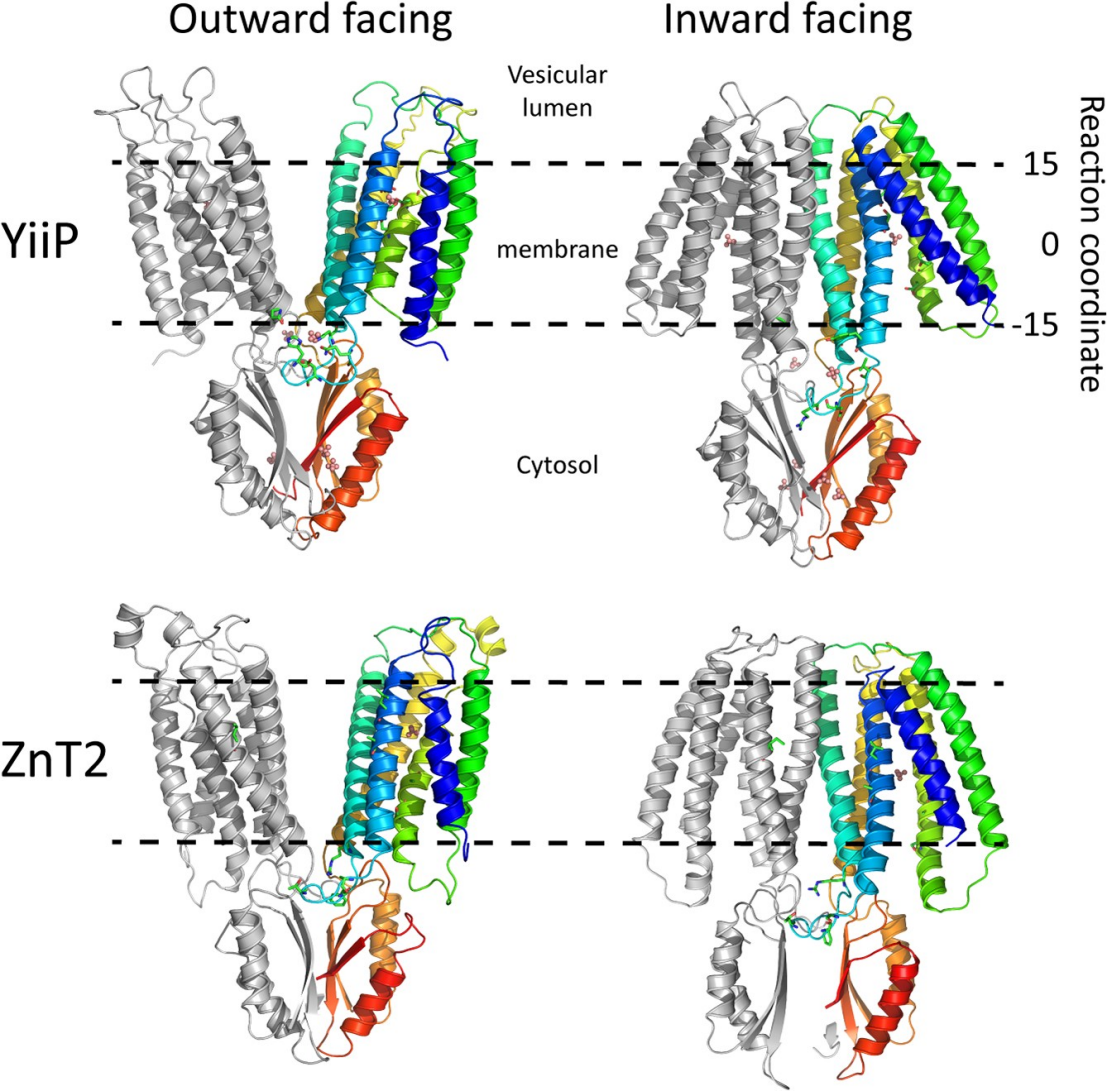
3D structures of YiiP and ZnT2. Side view of YiiP and ZnT2 3D models in the OF and IF conformations. The membrane is shown as dashed lines, the zinc ions are shown as pink spheres, and the zinc-binding sites residues are shown in licorice representation. One monomer is colored in rainbow colors. The reaction coordinate is the z-axis (see **Fig 2**).

The ZnT2 models were constructed using the X-ray structure of the outward-facing (OF; [23]) and the cryo-EM structure of the inward-facing (IF; [24]) conformations of YiiP (**Fig 1**), based on their respective sequence alignments by satisfaction of spatial constraints (**S1 Fig**) using the Memoir [25] modeling suite. Memoir is specifically designed for transmembrane proteins and performs better than HHpred [26] and Swiss-Model [27]. The quality of the models was verified using Verify3D, providing a global and local assessment of the correctness of fold structures at the residue level. We found that Memoir models have better Verify3D scores than HHpred models and the scores were further improved after refinement with ModRefiner [28]. Accordingly, four systems constructed with Memoir and minimized with ModRefiner were chosen for the computations. We then minimized their energies and equilibrated them using the Molaris simulation package [29,30]. All systems were stable as manifested by low RMSD values to their initial coordinates (less than 2Å for the backbone atoms).

Following the construction and equilibration of these models and considering the expected homodimeric nature of ZnT2 [13,31], we initially explored the possible location of the zinc permeation pathway along the dimerization interface, as previously suggested for YiiP [32]. However, a detailed examination of the structures and models revealed that there are almost no polar residues present along the dimeric interface, and in the OF conformation the monomers are not close enough to form a polar pore to stabilize zinc ions. In this respect, the bacterial Na^+^/H^+^ antiporter also functions as a dimer, whereas its sodium translocation pathway is located within each monomer and not between them [33–35]. We therefore searched for a highly polar zinc permeation pathway located within each YiiP/ZnT2 monomer and not between them. Consistent with previous studies, the X-ray structure of the bacterial YiiP monomer reveals a cavity leading from the zinc binding site to the extracellular milieu, lined with many polar and negatively-charged amino acid residues. However, the cavity grows very wide, and we therefore explored our models for several putative entry and exit routes and zinc permeation pathway.

Prior to computing the binding energies of the zinc ion, we sought to study the protonation state for the binding site residues (site A, see **Fig 1**and [23]), harboring Asp and His residues. Determining the most stable protonation state is critical to attain correct binding energies of the ion because the charge distribution has arguably the biggest contribution to the interaction between the transporter protein and its zinc substrate. To that end, we calculated the relative energies of the different protonation states for YiiP and ZnT2 using the Molaris package (see Methods for more details). The results are summarized in **S2 Table**, and the zinc binding calculations (see below) were conducted using the lowest-energy protonation state. As a control, we performed the binding calculations for several other protonation states for the OF YiiP structure and ZnT2 model, and the curves were very similar qualitatively, but showed a gradual difference quantitatively as the net negative charge increases, mostly around zinc binding site A (**S2 Fig**). This hints at the interaction between protons and zinc ions, where protonation of side-chains weakens the binding of zinc ions, as expected for a putative proton-coupled zinc exchanger. Further investigation of the coupling between the protonation state and the translocation of zinc ions is beyond the scope of this work and will be explored thoroughly in a subsequent study.

### Binding energies in the zinc permeation pathway

**Fig 2:**
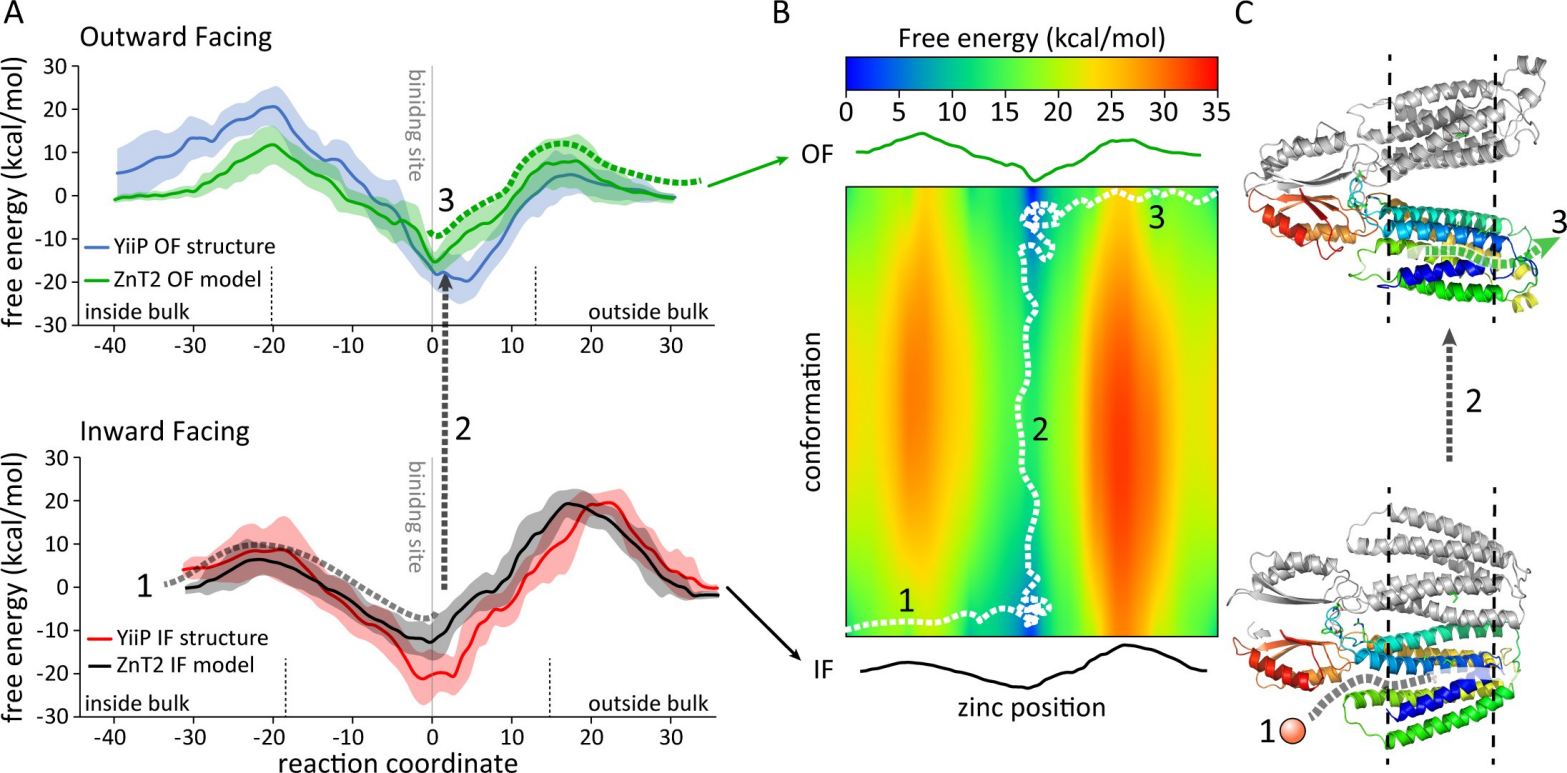
Free energy calculations of zinc-binding along the putative YiiP and ZnT2 permeation pathways. (A) Free energy of hydration for zinc in YiiP and ZnT2. The zinc-binding site (site A) is marked by a vertical line and the bulks are labeled. Each line represents the running average of several paths and starting structures (see methods), and the faded contour represents the SEM. The reaction coordinate is the distance (in Å) from the binding site, in the direction of the bulks. (B) The 2D energy landscape, using as coordinates the position of the zinc and the conformational change, the intermediate energies are computed as a linear interpolation of the IF and OF energies from (A) and the purpose is to provide a visual aid to the system’s energy landscape. (C) Model structures of ZnT2 exemplify the reaction coordinate for the zinc ion. In (A), (B) and (C), a pseudo random path depicting a zinc ion traversing the transporter is shown in dashed lines, split into 3 sections (see *Exploration of the zinc permeation pathway* in the Results and Discussion section).

The zinc binding energy profile for the most stable protonation states (see above) on the equilibrated structures of ZnT2 and YiiP are depicted in **Fig 2A**. The binding energies were calculated using the Molaris PDLD/S-LRA method developed and refined over the years by the Warshel group [36,37] and proven valuable in numerous studies starting over 35 years ago, exploring various and diverse biological systems [29,37,38]. The curves were produced by averaging over several pathways selected along the cavities, where the MD simulations allow extensive sampling for the zinc ion position as well as the local conformation of the transporter protein (see the Methods section for more details). We computationally explored several alternative permeation pathways based on the volume available in the open-direction cavity (outward in the OF conformation and inward in the IF conformation, converging at site A). For the cavity on the closed side, we extrapolated the positions based on residues interacting with the zinc ion in the opposite conformation, as well as inspecting the conformational change in the CG and morph trajectories (see below).

**Fig 3:**
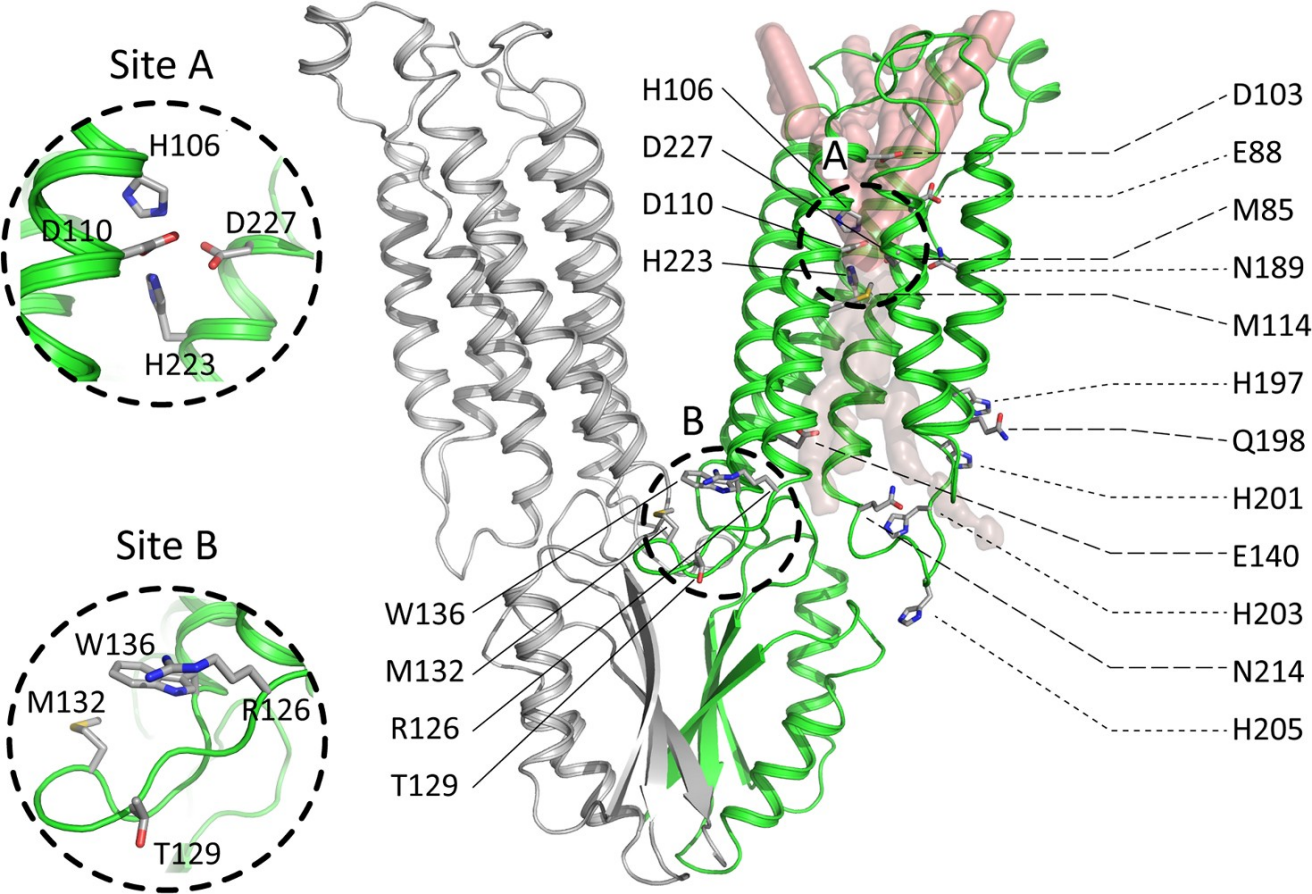
The putative transmembrane zinc permeation pathway. ZnT2 OF model is shown in green; the zinc permeation pathways explored are shown as translucent volumes. The pathways going out represent plausible zinc pathways (see **Fig 2**energies), whereas the pathway going inside the cell represent a pathway that is too high in energy (faint pink), requiring the conformational change to OF for the zinc to pass through. The equivalent pathways for the IF model are shown in **S6 Fig**. Binding sites A and B [23] are marked by dashed circles and a zoom-in view is presented to the left. The residues mutated in this study are shown in licorice representation and are labeled.

The energy curves of the OF conformation revealed a strong zinc-binding at the known binding site (site A in **Fig 3**and [23]), as would be expected for a zinc transporter (corresponding to an affinity in the pM-nM range, see note regarding quantitative evaluation at the end of this subsection). Although there are no published experimental K_d_ values for zinc binding *per se*, in the study of Chao and Fu [39] on the *E*.*coli* zinc transporter ZitB (25% sequence identity to ZnT2), zinc translocation is composed of a two-steps process: a relatively rapid binding of zinc followed by a rate-limiting step in which the transporter undergoes conformational changes. Our energy profiles suggest a qualitatively similar pattern. Their kinetic study on ZitB reveals K_M_ values in the high μM range. However, this discrepancy can be attributed to different functions and properties of ZitB and ZnT2, as well as possible deviations between the value of K_M_ and K_d_. In this respect, the abundant intracellular zinc binding protein metallothionein (capable of transferring zinc to the apo-forms of zinc-dependent enzymes and presumably to zinc transporters as well) displays a K_Zn_ of 3.2x10^13^ M^-1^ (i.e. a high metal binding constant), hence being consistent with the predicted concentration range [40,41].

Moving further along the energy curves, from the binding site to the open side direction, the binding energy steadily increases, albeit with relatively small barriers, as the interactions between the binding site residues and zinc are weakening, until a plateau is observed towards the bulk. To ensure that the suggested permeation pathway allows for selective zinc transport via alternating access, we additionally computed the energy profile in the closed direction. Indeed, **Fig 2A** shows high energy barriers for ions on the closed side of the transporter, compared to the open side, typical for alternating access. We then repeated this process for the IF conformations. Here we qualitatively obtained curves similar to the ones for the OF conformations, but as mirror images. The selection of the permeation pathway was not as obvious as above, because the cavity was not as large and visually apparent. Thus, we generated several putative trajectories for the conformational transition from OF to IF and examined very carefully the changes between one conformation and the other, i.e. where do these cavities form, and which interactions are disrupted. We produced the trajectory using two strategies: (i) our newly developed CG normal mode MC simulation (see the Methods section); and (ii) a Cartesian morph. Although these trajectories are not guaranteed to represent the precise physiological conformational change, we found them very instructive and were able to suggest several pathways, albeit more tightly packed than in the OF paths. Our energy calculations support the suggested permeation pathways, as they represent expected curves for zinc ion translocation across a transporter: low energies for the binding site, with barriers on both sides and a higher barrier on the closed side (**Fig 2A**). In **Fig 2B** we show the energy landscape for the zinc ion as a function of its position within the protein and the ZnT2 conformation (using the OF and IF end points). We estimated the conformation change barrier roughly at ~16 kcal/mol based on the ~1.5 sec^-1^ rates reported for YiiP [42]. Walking along the energy landscape (**Fig 2B** and **S1 Movie**), we divided the path into three sections for clarity; the zinc ion enters the IF conformation from the cytoplasm and binds at the binding site (site A [23]; section 1); then, ZnT2 undergoes a conformational change to the OF state (section 2), and finally the zinc ion exits to the vesicular lumen (for ZnT2) or to the extracellular milieu (for YiiP; section 3). In this model, the transport of the zinc ion along the membrane necessitates a conformational change of the transporter protein, since the IF or the OF conformations alone harbor a high energy barrier. As mentioned above, coupling zinc translocation to the proton gradient and the directionality will be explored in a subsequent dedicated study.

Since the energy profiles are a crucial component of the current study, we sought to assess their convergence. To validate the robustness of our methods and models and to prove proper sampling for the position of the ion and the local conformation of the interacting protein residues at each point, we chose the ZnT2 OF system as a control, and performed the same calculations using 10-fold longer simulations. The results obtained were essentially the same regardless of simulation length (see **S3 Fig**), indicating that our calculations most probably converged within the original simulation times used. We wish to emphasize that the validity of our calculated permeation pathway is reinforced by the experimental mutational analysis and their consequences presented below. Therefore, the precise qualitative nature of the binding curves found in the current study (e.g. the K_d_ we obtained and the conformational change energy) are prone to uncertainty resulting mainly from the modeling process as well as limitations of the computational methods (e.g. simulating a single monomer, not considering the probability average of all the different protonation states that are slightly higher in energy). Thus, the computational results and conclusions of this work are likely to be correct, while the calculated values should be still considered as a qualitative trend rather than actual quantitative numbers. The consistency of the calculated results with the observed mutational experimental analyses below supports the acceptance of this mode of calculation as a proper representation of the functional key residues along the putative zinc permeation pathway.

### Predicting key residues along the zinc permeation pathway

To provide functional validation to our proposed zinc permeation pathway, we next aimed at predicting the amino acid residues that might play a functional role in zinc permeation. We searched for residues that contributed significantly to the calculated binding free energy (see **Fig 2**) along the permeation pathway, in several trajectories, and in several positions. We note that considering stabilizing interactions at the energy barriers is important as well, because they lower the barrier on the open-side of the transporter. Notably, one of the binding sites revealed by Lu et al., (site B; [23]) does not appear to directly participate in the zinc permeation pathway. Although this site appears to be sufficiently close to the permeation pathway to participate, a careful examination of the CG and morph trajectories strongly indicates that this is not the case. Site B is deformed in the IF conformation, and its residues are too far from the putative permeation pathway that we suggest. In this context, we propose that site B may act as a ‘waiting-area’ for zinc ions; that is, zinc ions initially bind to site B and are then translocated to the zinc permeation pathway. This might prove functionally crucial since zinc is found at very low intracellular concentrations, and it is bound and shuttled by zinc-binding proteins such as metallothioneins [9,10]. Thus, based on our computations and models we hypothesize that an auxiliary binding site to capture zinc when the transporter is undergoing conformational changes (i.e. during the transport cycle) might render a more continuous and robust ion flux. This too will be further investigated in dedicated future studies.

Interestingly, according to ZnT2 modeling, the residues that participate in zinc ion binding (site A) are very similar in ZnT2 (two Asp and two His) and in YiiP (three Asp and one His, see **S1 Fig**). In support of this suggestion, Hoch et al., previously showed [42] that these differences between YiiP and ZnT5 or ZnT8 sequences contribute to the selectivity of the transporters towards zinc as a substrate, compared to YiiP which transports cadmium and zinc [42]. After careful consideration of the residues interacting with the zinc ion along the putative permeation pathway, we considered their evolutionary conservation (see **S1 Fig**) and assembled a list of candidate residues that are both important for zinc interaction and hydration, and are also conserved. This was done following the rationale that evolutionary-conserved residues are more likely to be functionally important. Herein, the residues studied by site-directed mutagenesis, shown in **Fig 3,** were carefully selected according to their evolutionary conservation and their calculated energy contribution to zinc binding.

### Site-directed mutagenesis of key residues along the zinc permeation pathway disrupts ZnT2 transport activity

**Fig 4:**
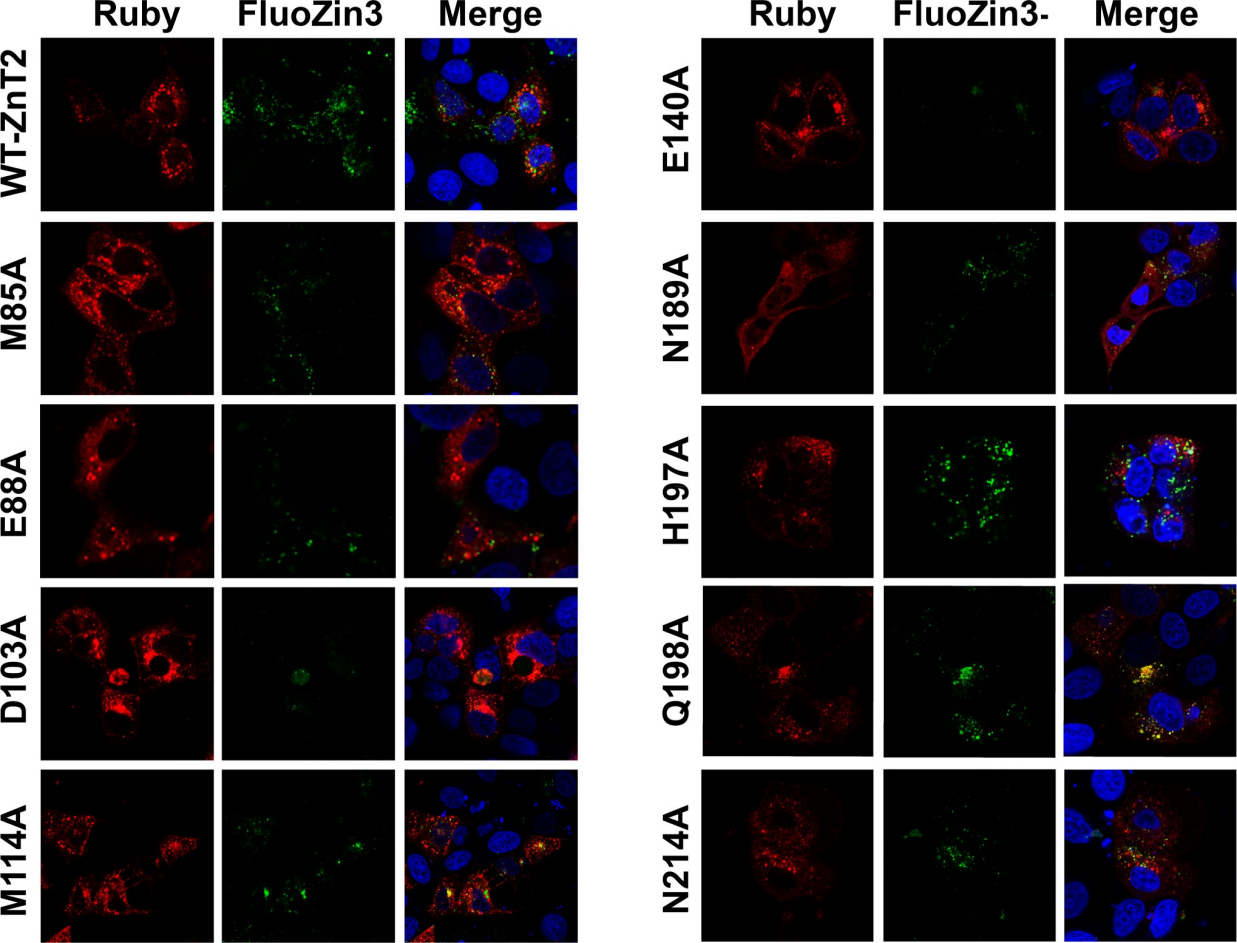
Site-directed mutagenesis at key residues of the zinc permeation pathway disrupting zinc transport activity of ZnT2. FluoZin3 (green fluorescence) indicates zinc accumulation. Red fluorescence shows the ZnT2-Ruby protein of the WT and ZnT2 mutants as indicated at the left column. Hoechst 33342 (blue fluorescence) was used to fluorescently label nuclei. A magnification of ×63 under immersion oil is presented.

To provide experimental validation for the computational analyses delineating the putative zinc permeation pathway of ZnT2, we mutated selected residues and assessed their impact on actual zinc transport activity in live human cells. While employing Ruby-tagged ZnT2 expression plasmids (emitting red fluorescence), the green fluorescence of the selective zinc probe FluoZin 3-AM, was used. Hence, zinc-containing vesicles were detected as green fluorescent vesicles solely in cells transiently transfected with an active ZnT2 transporter after incubation with zinc as previously described [19] **(Fig 4)**.

**Fig 5:**
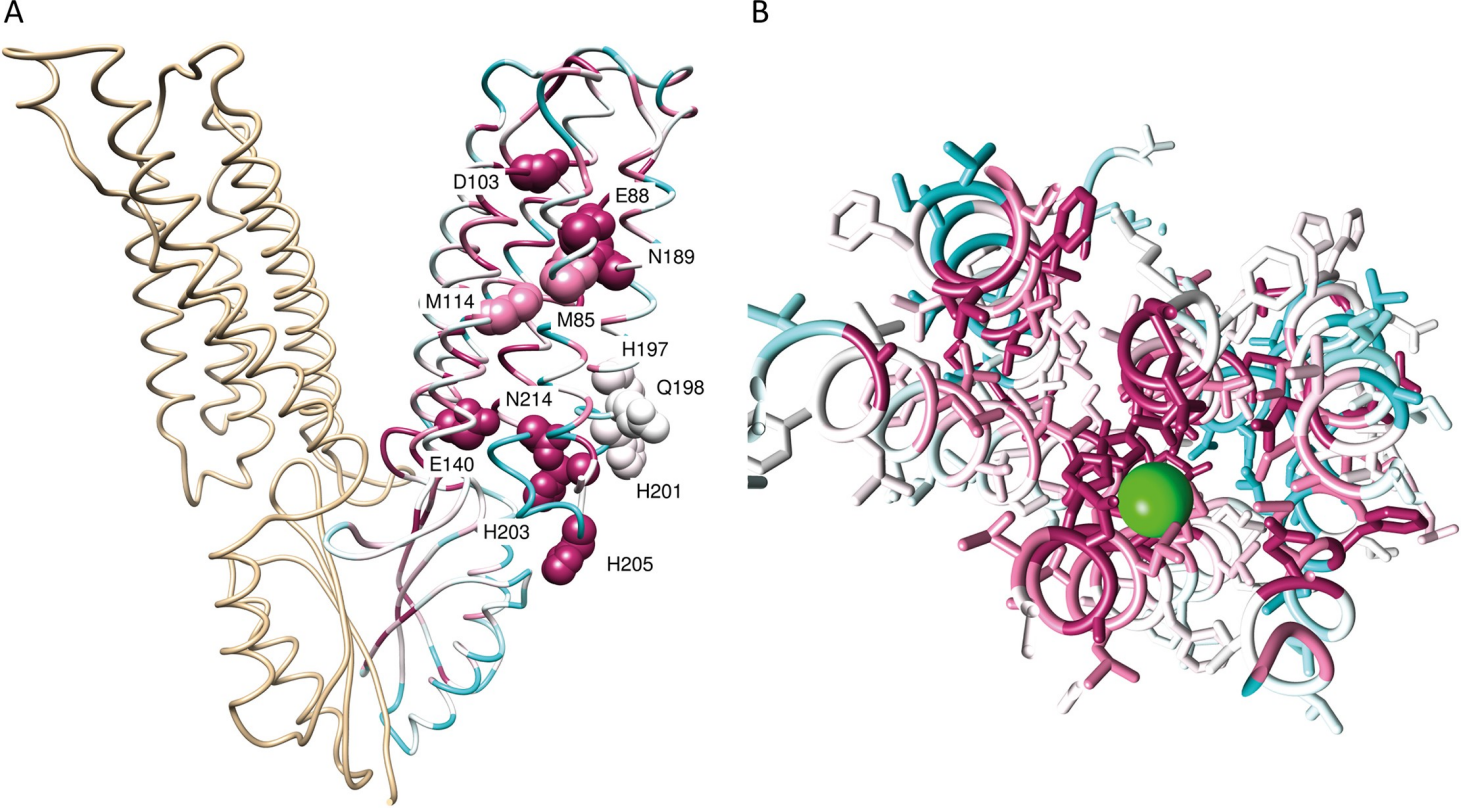
Evolutionary conservation. The ZnT2 model color-coded by ConSurf evolutionary conservation score. (A) Side view of ZnT2 dimer model that was generated based on alignment to YiiP structure (3H90) with all point mutations studied (shown in CPK and labeled). Only one monomer is colored by ConSurf showing conservation scores, while the second copy, superimposed to its YiiP counterpart, is colored in tan. (B) Top view of the putative zinc permeation pathway along the ZnT2 transmembrane region. The zinc ion is colored in green and placed on the equivalent crystallographic location of the zinc ion in YiiP.

Our choice of specific protein residues to study was made by energetic, structural, and evolutionary conservation basis, as explained above. **Fig 5B** shows a ZnT2 monomer color coded by conservation from the extracellular side of the membrane. Only residues located at the transmembrane domain and with the highest degree of conservation (9) and the lowest (1 and 2) are shown for clarity. This panel shows that the vast majority of highly conserved residues face the internal pore of the transporter, while the fast-evolving residues are facing the lipid core of the membrane. This fits the model fold and the expected biological function of ZnT2, where functional conserved residues lining the permeation pathway cannot tolerate evolutionary changes, while more rapidly evolving hydrophobic residues facing the membrane are able to accommodate a range of hydrophobic residues without impacting the membrane-protein interaction. Therefore, the positions we chose have an important energetic contribution to zinc binding, are highly conserved, face the pore according to our structural model, and in consequence, are expected to impact zinc binding.

The results of the site-directed mutagenesis are summarized in **Table 1**. Additionally, we computed the evolutionary conservation of the ZnT2 family. **Fig 5**shows that most mutated positions are highly conserved (in purple) and face the putative zinc permeation pore (**Fig 5**), with the exception of H197 and Q198 which are slightly variable. All mutants displayed similar or even higher red fluorescence levels (Ruby fluorescence) compared to the WT-ZnT2, indicating the proper expression of these ZnT2 mutants **(Table 1,** right column**)**. For our analysis, we grouped mutated positions according to their physicochemical, biological and evolutionary conservation relevance, and hence in this way their biological impact and phenotypic characteristics could be more easily understood (notice that the groups are not necessarily exclusive). The first group includes residues E88, D103 and E140, which are likely involved in direct interaction with zinc; these residues are negatively charged, highly conserved and located along the putative zinc permeation pathway according to the proposed 3D model. Indeed, site-directed substitution of these residues to Ala proved to be highly deleterious for ZnT2 function with about 90% loss of WT ZnT2 zinc transport activity (**Table 1**). Thus, we considered these positions to be important for zinc transport. Notably, whereas the E140A mutant showed a low but significant 20% decrease in the overall number of ZnT2 vesicles when compared to the WT ZnT2, the decrease in its zinc transport function was much more profound. The second group includes residues M85, M114, N189 and N214; these residues are conserved and are spatially located below and above the zinc-binding site with respect to the membrane plane (**Fig 5**). However, in contrast to the first group, this group of residues showed a moderate impact on zinc transport, upon substitution to Ala, with 24–52% decrease in zinc transport capacity (**Table 1 and Fig 4**). This suggests that the impact of polar residues along the permeation pathway on binding and/or transport of zinc is manifested collectively, as the contribution of each single residue to zinc permeation is smaller compared to the charged residues aforementioned. We also observed an additional impact on ZnT2 function; for example, E88A and N189A displayed loss of vesicular localization (**Fig 4**) and furthermore, M114A, E140A and N214A exhibited a decreased number of vesicles per cell when compared to the WT-ZnT2 (**Table 1**). Supporting our findings and applying a different experimental setup, a recent study on the closely related ZnT1 revealed that ZnT1’s equivalent of ZnT2’s mutants, E88A and N189A, rendered ZnT1 dysfunctional [43]. Our findings suggest that these five residues (E88, M114, E140, N189, and N214) have a significant role in protein structure and stability in addition to their role in zinc binding and zinc permeation. In contrast, D103A displayed a high number of ZnT2 vesicles while showing very little zinc accumulation, suggesting that this residue has an important role in zinc transport with little impact on transporter stability and subcellular localization. The third group of mutants including M85A, H197A and Q198A retained the canonical subcellular vesicular localization (**Fig 4 and Table 1**). In this respect, H197 and Q198 are not evolutionary conserved residues, are located near the cytoplasmic region, and point away from the zinc permeation pathway (**Fig 5B**), according to the proposed structural model. Hence, they were not expected *a priori* to impair zinc transport. Indeed, H197A and Q198A retained 80–90% of WT-ZnT2 zinc transport activity, with no significant differences when compared to zinc accumulation mediated by WT ZnT2 (**Fig 4 and Table 1**).

**Table 1:** The impact of different mutations on zinc accumulation and ZnT2 localization.

Amino acid substitution	Zinc accumulation- FluoZin 3-AM intensity (% of WT)	% of the number of vesicles/cell compared to WT	Main subcellular localization	ZnT2 expression-Ruby fluorescence intensity (% of WT)
WT-ZnT2	100	100	Vesicular	100
M85A	*76	83	Vesicular	126
E88A	*16	**60	Non-vesicular	*107
D103A	*11	84	Vesicular	*144
M114A	*48	*72	Vesicular	*89
E140A	*10	**79	Vesicular	97
N189A	*62	**47	Non-vesicular	*148
H197A	90	96	Vesicular	100
Q198A	80	90	Vesicular	117
N214A	*56	**72	Vesicular	*94
His201-203-205Gly	86	89	Vesicular	*99
RFP Empty vector	*11	*2.9	Non-vesicular	*41

* Indicates that these values were statistically different (*p<0*.*05*) from cells transfected with the WT-ZnT2.

** Indicates that these values were statistically different (*p<0*.*01*) from cells transfected with the WT-ZnT2.

We also focused on a fourth group including residues H201, H203, and H205, which are part of the conserved GHGHSH motif (His-rich loop) located between TM helices IV and V in ZnT2 but are not present in the bacterial homologue, YiiP. This motif was previously suggested to be involved in sensing cytosolic zinc levels [44,45] or in mediating the activation of tissue-nonspecific alkaline phosphatase (TNAP) by ZnT5 [46]. However, based on our model of the human ZnT2, all three His residues are pointing away from the central permeation pore and are positioned in close proximity to the exit of the putative permeation tunnel. In different initial structural models obtained for ZnT2, these three His residues were in very different conformations, since the GHGHSH motif is a highly charged flexible loop and lacks complete template information (see **S1 Fig**), thus had to be modeled *ab initio* by FREAD in Memoir suite. In most models, these three His residues face the water bulk or other peripheral residues and their high conservation and positive charge suggests a role in the stabilization of ZnT2 with acidic phospholipid head groups or an allosteric zinc regulation role, rather than direct interaction with the transported zinc ion. Indeed, substitution of all three His residues to Gly did not exert any deleterious effect on the zinc transport capacity of ZnT2. This further suggests that the GHGHSH motif in ZnT2 is not directly involved in zinc translocation to site A and across the transporter, *i*.*e*. there are no direct interactions between the zinc ion (or its first hydration shell water molecules) and the histidine side chains of the GHGHSH motif. A very recent publication strengthen our findings, showing that deletion or substitution of these His residues to Ala in ZnT2 did not affect zinc transport activity [71]. However, an allosteric regulatory role of this motif cannot be excluded.

In summary, site-directed mutagenesis of seven key residues along the putative zinc permeation pathway of ZnT2, markedly impaired its zinc transport function. In contrast, site-directed mutations at non-conserved residues (H197A and Q198A), and at the conserved GHGHSH motif that is suggested to point away from the zinc permeation pathway, had only a minor deleterious impact on the zinc transport function of ZnT2. Taken together, residues predicted by our model and free-energy calculations, supported by the conservation analysis, agree well with our experimental validation of ZnT2 functionality.

## Discussion

In this study we demonstrated the power and potential of multiscale approaches by analyzing the zinc permeation pathway of ZnT2, highlighting the constructive synergism of computations and functional validation experiments. The principles of multiscale modeling used in this study, include representing the model structure using a CG model with a reduced number of atoms. Such approaches are highly advisable to accelerate a simulation (by reducing the degrees of freedom) and to perform calculations that would otherwise be extremely challenging (such as NMA). The binding free-energy calculations were also performed with a semi-macroscopic method, which is a form of reduced-dimensionality modeling, allowing faster convergence and thus more stable results. The reader is directed to other studies presented in this special issue, regarding other various multiscale approaches and techniques.

More specifically, in the current study we undertook extensive CG simulations of our proposed model of ZnT2, which provided structural and evolutionary information which delineates, for the first time, the putative zinc permeation pathway of ZnT2, from the cytoplasm into the lumen of intracellular vesicles (or the extracellular milieu in the case of YiiP). This proposed permeation pathway harbors one central zinc binding site (site A [23] and **Fig 3**) and two cavities showing alternating-access in the two principal conformations of YiiP and ZnT2 and possibly in other zinc transporter homologues as well (**Fig 2**). To functionally validate this permeation pathway experimentally, we performed site-directed mutagenesis to target various residues along the zinc permeation pathway (**Fig 4**).

Our rationale was to try to properly predict, based on the YiiP crystal structure and homology-based model of ZnT2, the correct deleterious impact of site-directed mutagenesis of key residues along the permeation pathway on the zinc transport activity of ZnT2 in viable cells. Indeed, this process lent strong experimental support to our structure modeling and zinc permeation pathway prediction.

Interestingly, a recent cryo-EM structure of the ZnTB zinc transporter was revealed [47], showing a pentameric architecture. ZnTB is proton-driven, precisely as ZnT2 is considered to be, however their sequence similarity is very low, and therefore ZnTB was not deemed a suitable template for ZnT2 in our present work.

We selected residues facing towards the putative permeation pathway with high contribution to the calculated zinc binding energy and used conservation analysis as a cross validation prior to the mutagenesis. Indeed, all the conserved residues that we computationally predicted to be important for zinc permeation, experimentally impaired the zinc transport function of ZnT2 upon substitution to alanine, while five of these mutants exhibited proper vesicular localization. As a negative control, we showed that residues that were facing away from the zinc permeation pathway including H197A, Q198A, and the GHGHSH motif, or having a tendency towards a less organized secondary structure (i.e. disordered region), showed a minimal deleterious effect on zinc transport activity of ZnT2, hence further supporting our hypothesis.

Thus, multiscale computational analyses complemented with functional experimental validation lead to the construction of valid models for the 3D functional conformations of human ZnT2 and the zinc translocation pathway. Furthermore, we were able to successfully predict important residues around the putative binding site involved in zinc binding and translocation, both in the direction of the cytoplasm and the extracellular milieu. From a translational medicine perspective, such zinc permeation pathway studies may facilitate the screening and identification of small molecules that can correct the proper folding and/or function of mutant transporter proteins.

In this respect, pharmacoperones are recently emerging as a novel class of hydrophobic small molecules that can bind to mutant misfolded and inactive proteins, thereby restoring their proper folding, subcellular sorting, and function [48,49]; this novel approach is currently known as pharmacoperone drug therapy. These mutant proteins which are associated with various human disorders include enzymes, receptors, channels, and transporters [48,49]. For example, Menkes disease is a neurodegenerative disease presenting with seizures, lethargy and hypotonia, resulting in death in early childhood. This disorder is caused by copper deficiency due to mutations in *ATP7A* that encodes for the copper-transporting ATPase 1 transporter [50]. Copper-transporting ATPase 1, which is localized in the *trans*-Golgi network, functions as an ATP-driven intracellular pump that transports copper into the *trans*-Golgi network for incorporation into copper-requiring enzymes; whereas, those pumps that are localized on the plasma membrane are involved in copper transport out of the cell [51]. Some of the mutant copper-transporting ATPase 1 transporters are misfolded and hence retained in the ER [50]. These mutants can be corrected by pharmacoperones, further supporting the suggestion that the ER-retained mutant is misfolded [50]. *In vitro*, copper supplementation of the culture media results in the correction of the mislocalization [50]. Therefore, the substrate for the transporter serves as a pharmacoperone, facilitating the folding of the mutant protein. In Menkes patients with residual copper-transport activity, early treatment with copper injections can normalize clinical outcomes and increases survival [52]. Hence, it is possible that such pharmacoperones could be identified for mutant zinc transporters like ZnT2 and other zinc transporters including ZIP4 that can correct the proper folding, subcellular sorting, and zinc transport, thereby providing a targeted therapeutic strategy to overcome well-defined diseases associated with zinc deficiency or other solute transport deficiencies. We hope that our present study will enhance our understanding of zinc transporters towards this strategy, and believe that the approach presented herein will convince others to consider multiscale modeling and experimental complementation as a platform to address pressing biomedical issues.

## Methods

### Construction of the 3D models

ZnT2 models were generated based on the OF and IF structures of *E*. *coli* YiiP (PDB: 3H90 and 5VRF, respectively). Modeling of ZnT2 was carried out by Memoir method [25], specifically designed to exploit the structural constraints imposed by membrane proteins. Briefly, homologous sequences are aligned by MP-T [53], guided by the membrane information from iMembrane [54]. Membrane information is again used in model building by the MEDELLER program, designed specifically to construct homology-based membrane protein core structures [55]. Finally, the model built by MEDELLER is completed with a membrane protein-specific version of the FREAD loop-modelling method [56]. All homology models were refined and minimized by ModRefiner [28]. All models had similar Verify3D profile average scores [28]. Alignment of the ZnT2 model to the YiiP structures used one point per residue (the CA atom). For visualization purposes, **Fig 3**fitting was performed using only the C-terminal domain residues, whereas **Fig 5**fitting was performed using all residues.

### Simulation system

The systems for YiiP and ZnT2, both in the IF and OF conformations, were constructed as follows: the transporter homodimer was inserted into a 30Å-thick 3D particle grid emulating a hydrophobic membrane, using the Molaris software package [29,30]. The gap between the protomers was treated as part of the membrane milieu, as this gap exists within the expected membrane space and it is suggested to be non-functional [24]. Then, the systems were hydrated with a 40Å-radius water sphere, using explicit 3-particle water molecules, and were submitted to energy minimization using the steepest descent algorithm followed by a short local relaxation simulation of 100 ps. Relaxation was performed in the presence of a zinc ion at the putative binding site (based on the structures of 3H90 and 5VRF), to prevent charge repulsion between the binding site residues.

### Binding energy calculations

To obtain the binding energy curves, the zinc ion at site A was vertically moved by 1Å intervals at the z-axis, to either side of the membrane, until it reached a pre-selected point in the water, i.e. the extracellular (vesicular lumen) or intracellular bulk. For each such position of zinc, 10 PDLD/S-LRA calculations (see below) were performed entailing an additional short step of relaxation of 10–20 ps, allowing the transporter to relax around the new position of the zinc ion, and to generate different starting configurations. Being a semi-macroscopic method, with CG elements, the PDLD/S-LRA method converges substantially faster than umbrella sampling/PMF methods, and calculations using typically these parameters have been successfully applied to many complex biological systems (e.g. [3,5,29]), regardless, we performed longer simulations as a control for one example (see Results and **S3 Fig**). During the relaxation process, the zinc ion could freely move in the xy-plane. This treatment reduced as much as possible the bias of selecting the path manually. For the open direction, where the cavity was very wide, we selected 5–7 such trajectories (i.e. different final points in the bulk), whereas only 3–4 trajectories were selected in the closed direction. Along the trajectory in the closed direction we computed short distances between the zinc ion and the transporter that are most probably non-physiological. These tight spots result in high-energy barriers that suggest an ion does not bind at this position in the biological system, but the energy could still be estimated and reported using the computational methods described below.

The binding energy calculation for each position point was performed using the scaled semi-macroscopic Protein Dipoles Langevin Dipoles approach (PDLD/S) of Molaris [29]. Water in this method is represented semi-macroscopically by Langevin dipoles. The energy is the average of the charged and uncharged states, following the linear response approximation (LRA), scaled using a dielectric constant ε = 8 for the protein. Convergence was achieved by running molecular dynamics (MD) simulations for the relaxation and averaging the results of the different conformations (PDLD/S-LRA) [36]. The reported energy was the average of all repeats (n = 10) and for all trajectories (3–7; see above). Additionally, running averages were used in the full-atomistic PDLD binding curves, applying a window size of three, to better match the CG-nature of the system used for the conformational changes. The calculations were performed for several protonation states but only one protonation state is discussed in the main text based on the relative energies of the states (see below and see **S2 Fig**).

### Determination of the protonation state

YiiP and ZnT2 have 4 ionizable residues in binding site A (DDHD and HDHD, respectively). To assess which protonation state is the lowest in energy, we computed the total energy of the electrostatic cluster following the formalism presented previously [37,57]. The total energy of each state is given by the sum of: (i) the solvation energy of the ionized residues (representing the energy cost of bringing the ionized residues and the zinc ion from the bulk to the interior of the protein, relative to the system with zero charges); (ii) the energy of ionizing the given residues (His or Asp) in water, based on bulk pK_a_ values; (iii) the electrostatic energy between the ionized residues (Coulombic energy to bring the ionized residues from an infinite distance to their distance calculated in the binding site, using a dielectric of 80); and (iv) the electrostatic energy between the ionized residues and the zinc ion. We include the zinc ion to determine the protonation state energy because the states are used to compute the zinc binding energy curves. Therefore, we need to consider the most stable protonation state in the presence of zinc. **S2 Table** lists all the protonation states and associated values.

### Zinc ion parameters

The zinc ion was simulated using three different settings: (i) a single charged particle; (ii) a 7-particle entity arranged as an octahedral complex; (iii) and a 5-particle entity arranged as a tetrahedral complex, based on ligand-field theory [58]. Previous studies from the Warshel group showed that transition metals are better simulated when they are represented by an ion-and-ligands structure rather than a point charge. The partial charges as well as radii of the particles in the different models were calibrated such that the hydration energy of the zinc ion in water would match the literature value of -467 kcal/mol previously reported [21,22]. These settings in the protein yielded very similar results, thus we ultimately used the 5-particle entity for the reported energies in the present study.

### CG conformational change trajectory

For the conformational change MC trajectory, we used our newly developed method that will be described extensively in a future publication. In brief, we look for conformational transitions in the landscape of our simplified CG model [59,60], where the water is treated implicitly. To do so we consider first only the non-bonding and bonding interactions, evaluate the Cartesian second derivative and evaluate the corresponding Cartesian normal modes, which are then projected on the torsional angle (dihedral) space. Then we move along the torsional normal modes and generate a path from the initial to the final structure (in this case, the IF and OF conformations), using a Monte Carlo (MC) procedure with the Metropolis acceptance criterion. Finally, we evaluate the full CG energy along the MC generated path.

### Functional zinc transport assay of the different ZnT2 permeation pathway mutants in live cells

MCF-7 breast cancer cells were grown and transiently transfected with pcDNA3.1 zeo expression plasmids harboring WT ZnT2 or ZnT2 mutants tagged with a Ruby fluorescent protein as previously described [19,61]. Primers used for site-direct mutagenesis are listed in **S1 Table**. The empty vector of RFP was used as a negative control for zinc transport. Eighteen hours after transfection, cells were incubated for 1 hour in growth medium containing 75 μM ZnSO_4_ and then stained with a selective fluorescent zinc probe FluoZin3-AM (1μM). Cells were then analyzed using Aria IIIu-flow cytometer (for measuring median FluoZin3-AM and Ruby/RFP fluorescence), IN Cell Analyzer 2000 (for quantification of the number of vesicles per cell), or imaged by confocal microscopy as previously described [16]. Percentage of FluoZin3-AM fluorescence was calculated by dividing the median fluorescence intensity of FluoZin3-AM, as measured in the cells after transfection with the different mutant ZnT2 plasmids, by the median fluorescence intensity values of the WT ZnT2 protein. These values reflect the zinc transport activity of the different ZnT2 mutants compared to the WT ZnT2 (**Table 1**).

### Hypothesis testing

To test the hypothesis that zinc transport is lower in cells transfected with mutants ZnT2 as compared to cells transfected with WT ZnT2, we compared the median FluoZin3 fluorescence levels in cells transfected with ZnT2 mutants to the WT ZnT2, using one tailed T-Test with unequal variance. The hypothesis testing was followed by False Discovery Rate correction for multiple hypothesis testing with α = 0.05 [62]. The same statistical analysis was performed to test the hypothesis that Ruby fluorescence (indicating ZnT2 expression levels) is lower in cells transfected with ZnT2 mutants as compared to cells transfected with the WT ZnT2. **Table 1**shows the fluorescence intensity values as percent of WT for clarity; statistical analysis was conducted on the actual fluorescence levels of each mutant compared to the WT fluorescence.

### Computation of evolutionary conservation

Estimation of the evolutionary conservation for each protein position was computed using the ConSurf server [63–66].

### Molecular graphics and analyses

Performed with the UCSF Chimera package [67], VMD [68], and PyMOL [69].

## Supporting information

S1 Table Primers used to introduce mutations into ZnT2. (DOCX)

S2 Table Energy terms for protonation state relative energy. (DOCX)

S1 Fig Pairwise Sequence alignment. The pairwise sequence alignment between human ZnT2 and bacterial YiiP for the region modeled by Memoir. Residues are colored by the Zappo scheme [70]. TM annotation was obtained by iMembrane; T: Inner membrane (TM region facing lipid tails) H: Outer membrane (lipid polar heads) N: Non-membrane. 3h90 is the sequence of bacterial YiiP and ZnT2 the human ZnT2.
(EPS)

S2 Fig Protonation states zinc binding energies. PDLD/S-LRA binding curves similar to the ones presented in Fig 2 are shown for all protonation states explored in ZnT2 OF model. For visual clarity the SEM is shown for only one curve (gray contour) but have similar magnitudes in all the datasets.
(EPS)

S3 Fig Robustness of PDLD energy. PDLD/S-LRA binding calculations for the ZnT2 and YiiP OF model are presented using different simulation lengths, compared to the parameters used for this study (in black). See references [29,30] for more details on the different parameters. In brief, we extended the length of the relaxation (generating configurations; red), the energy calculation in water (to generate the reference energy; blue) and the energy calculation in protein (the binding step; green). n = 10. The SEM is shown for the production curve only (gray contour, same as **Fig 2**) for visual clarity, and has a similar magnitude in all the datasets.
(EPS)

S4 Fig Zinc settings tested for the study. The particles are shown as spheres and the force field bonds are shown as lines. The partial charge of each particle is indicated adjacent to it. The bond length is indicated by an arrow (only one bond length is shown for compactness, and all bond lengths are equal at each entity). The final setting used is the 5-particle one, highlighted in bold font. The angles are standard octagonal (90°) or tetrahedral (109.5°) and are not shown for compactness. The entities are presented at an angle resembling an isometric projection for clarity.
(TIF)

S5 Fig Expansion of the energy curves for all pathways explored in the ZnT2 OF conformation. The curves are an expansion of Fig 2. The SEM is shown for one path only (gray contour) for visual clarity and has a similar magnitude in all datasets. The different colors represent independent different trajectories.
(EPS)

S6 Fig The putative transmembrane zinc permeation pathway for the IF conformation. See **Fig 3**for details.
(TIF)

S7 Fig 2D energy landscape map for YiiP. Similar to the map shown in Fig 2B.
(EPS)

S1 Movie CG trajectory. An illustrative movie to highlight the conclusions of this work. The animation on the left shows the ZnT2 model, starting in the IF conformation. The pathways explored in Fig 2 are shown as a translucent volume, and the ion is shown in red. During the movie, the ion reaches the binding site and attaches to the putative residues (site A; shown in licorice). The movie then accelerates, and the conformational change occurs (on a much longer time scale than the binding). When the OF conformation is achieved, the zinc ion can detach and reach the vesicular lumen via one of the pathways explored for the OF conformation. The conformational changes shown were simulated using our CG-MC method (see methods) and the pathways were explored on the IF and OF static models (see Fig 2). The movement of the ion is along the positions used to generate the binding curves, on the static models as well, and is shown as an animation for visual purposes. The position of the membrane is shown as black lines. The movie is meant to serve as a visual aid and is not to scale (in the dimension of time). On the right we present the energy landscape map shown in Fig 2, with a tracker visualizing the state of the system in the energy landscape at that given time. The path traveled along the map is marked in a white line. The sporadic shakes of the tracker are for visual purposes only.
(MP4)
