# Correction: Demonstrating aspects of multiscale modeling by studying the permeation pathway of the human ZnT2 zinc transporter

**DOI:** 10.1371/journal.pcbi.1007021

**Published:** 2019-05-01

**Authors:** Yarden Golan, Raphael Alhadeff, Fabian Glaser, Assaf Ganoth, Arieh Warshel, Yehuda G. Assaraf

There is an error in reference 20. The correct reference is: Salusso A, Raimunda D. Pseudomonas Aeruginosa CDF Transporters CzcD and YiiP are Involved in Zn2+ Efflux, Outer Membrane Permeability and Antibiotic Resistance. Biophys J.; 2017; 112: 3, 16a. https://www.cell.com/biophysj/fulltext/S0006-3495(16)31152-3
