# Supplementary material for: Demonstrating aspects of multiscale modeling by studying the permeation pathway of the human ZnT2 zinc transporter
Source: PLoS Comput Biol. 2018 Nov 2;14(11):e1006503. doi: 10.1371/journal.pcbi.1006503 (PMC6241132; doi:10.1371/journal.pcbi.1006503)
Supplement: S1 Table — (DOCX) [file pcbi.1006503.s001.docx]

**Table S1. Primers used to introduce mutations into ZnT2**

| Name | Fw Sequence (5’ to 3’) | Rv Sequence (5’ to 3’) |
| --- | --- | --- |
| M85A-ZnT2 | TGCCATCTGCCTGTTGTTCGCGATCGGAGAAGTCGTTGGT | ACCAACGACTTCTCCGATCGCGAACAACAGGCAGATGGCA |
| E88A-ZnT2 | CTGTTGTTCATGATCGGAGCAGTCGTTGGTGGGTAC | GTACCCACCAACGACTGCTCCGATCATGAACAACAG |
| D103A-ZnT2 | AGCTTGGCTGTCATGACTGCTGCAGCACACCTGCTC | GAGCAGGTGTGCTGCAGCAGTCATGACAGCCAAGCT |
| M114A-ZnT2 | ACTGACTTTGCCAGCGCGCTCATCAGCCTCTTCT | AGAAGAGGCTGATGAGCGCGCTGGCAAAGTCAGT |
| E140A-ZnT2 | GGCTGGCAGAGAGCTGCGATCTTGGGAGCCCTG | CAGGGCTCCCAAGATCGCAGCTCTCTGCCAGCC |
| N189A-ZnT2 | GCTGCGCTGTGGCTGTGGCCATCATAATGGGGTTG | CAACCCCATTATGATGGCCACAGCCACAGCGCAGC |
| H197A-ZnT2 | TGGGGTTGACCCTTGCCCAGTCTGGCCATGGG | CCCATGGCCAGACTGGGCAAGGGTCAACCCCA |
| Q198A-ZnT2 | GGGTTGACCCTTCACGCGTCTGGCCATGGG | CCCATGGCCAGACGCGTGAAGGGTCAACCC |
| N214A-ZnT2 | AACCAGCAGGAGGAGGCCCCCAGCGTCCGAG | CTCGGACGCTGGGGGCCTCCTCCTGCTGGTT |
| His201-203-205Gly-ZnT2 | ACCCTTCACCAGTCTGGCGGGGGGGGCAGCGGCGGCACCACCAAC | GTTGGTGGTGCCGCCGCTGCCCCCCCCGCCAGACTGGTGAAGGGT |
| Q230A-ZnT2 | ATCGGCGACTTTATGGCGAGCATGGGTGTCCTAGTG | CACTAGGACACCCATGCTCGCCATAAAGTCGCCGAT |
