# Supplementary material for: Demonstrating aspects of multiscale modeling by studying the permeation pathway of the human ZnT2 zinc transporter
Source: PLoS Comput Biol. 2018 Nov 2;14(11):e1006503. doi: 10.1371/journal.pcbi.1006503 (PMC6241132; doi:10.1371/journal.pcbi.1006503)
Supplement: S2 Table — (DOCX) [file pcbi.1006503.s002.docx]

**Table S2. Energy terms for protonation state relative energy.**

| YiiP (OF) | | | | | | | | |
| --- | --- | --- | --- | --- | --- | --- | --- | --- |
| Residue charge | | | | Solvation energy | pK_a_ energy | Electrostatic energy between residues | Electrostatic energy from zinc ion | Total |
| H45 | D49 | H153 | D157 |  |  |  |  |  |
|  |  |  |  | reference | 0 | 0 | 0 | 0 |
| -1 |  |  |  | 0.67 | -4.34 | 0 | -3.16 | -6.83 |
|  | -1 |  |  | -0.22 | -4.34 | 0 | -2.97 | -7.53 |
|  |  | 1 |  | -1.12 | 1.26 | 0 | 3.48 | 3.62 |
|  |  |  | -1 | -0.51 | -4.34 | 0 | -3.05 | -7.90 |
| -1 | -1 |  |  | -1 | -8.68 | 0.93 | -6.14 | -14.89 |
| -1 |  | 1 |  | -1.58 | -3.08 | -0.94 | 0.319 | -5.285 |
| -1 |  |  | -1 | -0.21 | -8.68 | 1 | -6.22 | -14.11 |
|  | -1 | 1 |  | -0.99 | -3.08 | -1.04 | 0.51 | -4.6 |
|  | -1 |  | -1 | -1.6 | -8.68 | 0.83 | -6.02 | -15.46 |
|  |  | 1 | -1 | -0.5 | -3.08 | -1.09 | 0.43 | -4.23 |
| -1 | -1 | 1 |  | -1.84 | -7.42 | -1.05 | -2.65 | -12.97 |
| -1 | -1 |  | -1 | -1.51 | -13.02 | 2.77 | -9.19 | -20.95* |
| -1 |  | 1 | -1 | -0.37 | -7.42 | -1.034 | -2.73 | -11.55 |
|  | -1 | 1 | -1 | -0.6 | -7.42 | -1.29 | -2.54 | -11.85 |
| -1 | -1 | 1 | -1 | -1.74 | -11.76 | -0.3 | -5.70 | -19.51 |
|  | | | | | | | | |
| ZnT2 (OF) | | | | | | | | |
| Residue charge | | | | Solvation energy | pK_a_ energy | Electrostatic energy between residues | Electrostatic energy from zinc ion | Total |
| H106 | D110 | H223 | D227 |  |  |  |  |  |
|  |  |  |  | reference | 0 | 0 | 0 | 0 |
| 1 |  |  |  | -3.05 | 1.26 | 0 | 3.58 | 1.79 |
|  | -1 |  |  | -0.17 | -4.34 | 0 | -3.11 | -7.62 |
|  |  | 1 |  | -1.41 | 1.26 | 0 | 3.56 | 3.41 |
|  |  |  | -1 | 0.35 | -4.34 | 0 | -3.12 | -7.11 |
| 1 | -1 |  |  | -1.67 | -3.08 | -1.11 | 0.47 | -5.39 |
| 1 |  | 1 |  | -1.97 | 2.52 | 1.11 | 7.14 | 8.80 |
| 1 |  |  | -1 | -1.75 | -3.08 | -1.02 | 0.46 | -5.40 |
|  | -1 | 1 |  | -1.97 | -3.08 | -1.09 | 0.45 | -5.69 |
|  | -1 |  | -1 | -0.26 | -8.68 | 0.83 | -6.23 | -14.34* |
|  |  | 1 | -1 | -2.06 | -3.08 | -1.05 | 0.44 | -5.75 |
| 1 | -1 | 1 |  | -3.42 | -1.82 | -1.09 | 4.03 | -2.30 |
| 1 | -1 |  | -1 | -2.41 | -7.42 | -1.30 | -2.65 | -13.78 |
| 1 |  | 1 | -1 | -2.14 | -1.82 | -0.97 | 4.02 | -0.91 |
|  | -1 | 1 | -1 | -2.32 | -7.42 | -1.31 | -2.67 | -13.72 |
| 1 | -1 | 1 | -1 | -3.50 | -6.16 | -2.34 | 0.91 | -11.09 |

Table S2: Energy terms of the protonation states to determine which protonation state (site A) to use for the zinc binding calculations. All energies are in kcal/mol; empty cells indicate an uncharged residue; and the lowest energy is marked by an asterisk. See methods for more details.
