# Supplementary figures and images for: Demonstrating aspects of multiscale modeling by studying the permeation pathway of the human ZnT2 zinc transporter
Source: PLoS Comput Biol. 2018 Nov 2;14(11):e1006503. doi: 10.1371/journal.pcbi.1006503 (PMC6241132; doi:10.1371/journal.pcbi.1006503)

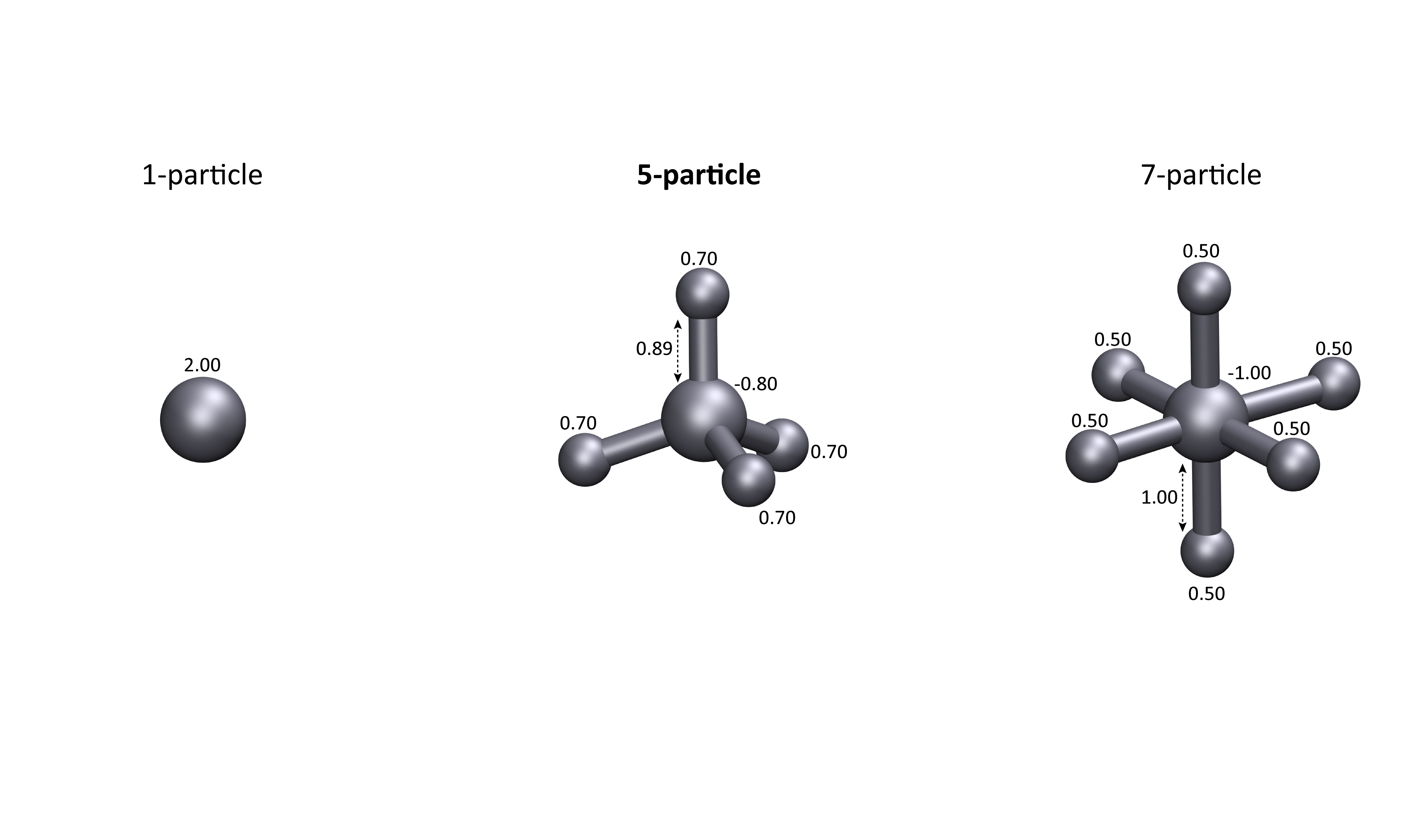

Supplement: S4 Fig — The particles are shown as spheres and the force field bonds are shown as lines. The partial charge of each particle is indicated adjacent to it. The bond length is indicated by an arrow (only one bond length is shown for compactness, and all bond lengths are equal at each entity). The final setting used is the 5-particle one, highlighted in bold font. The angles are standard octagonal (90°) or tetrahedral (109.5°) and are not shown for compactness. The entities are presented at an angle resembling an isometric projection for clarity. (TIF) [file pcbi.1006503.s006.tif]

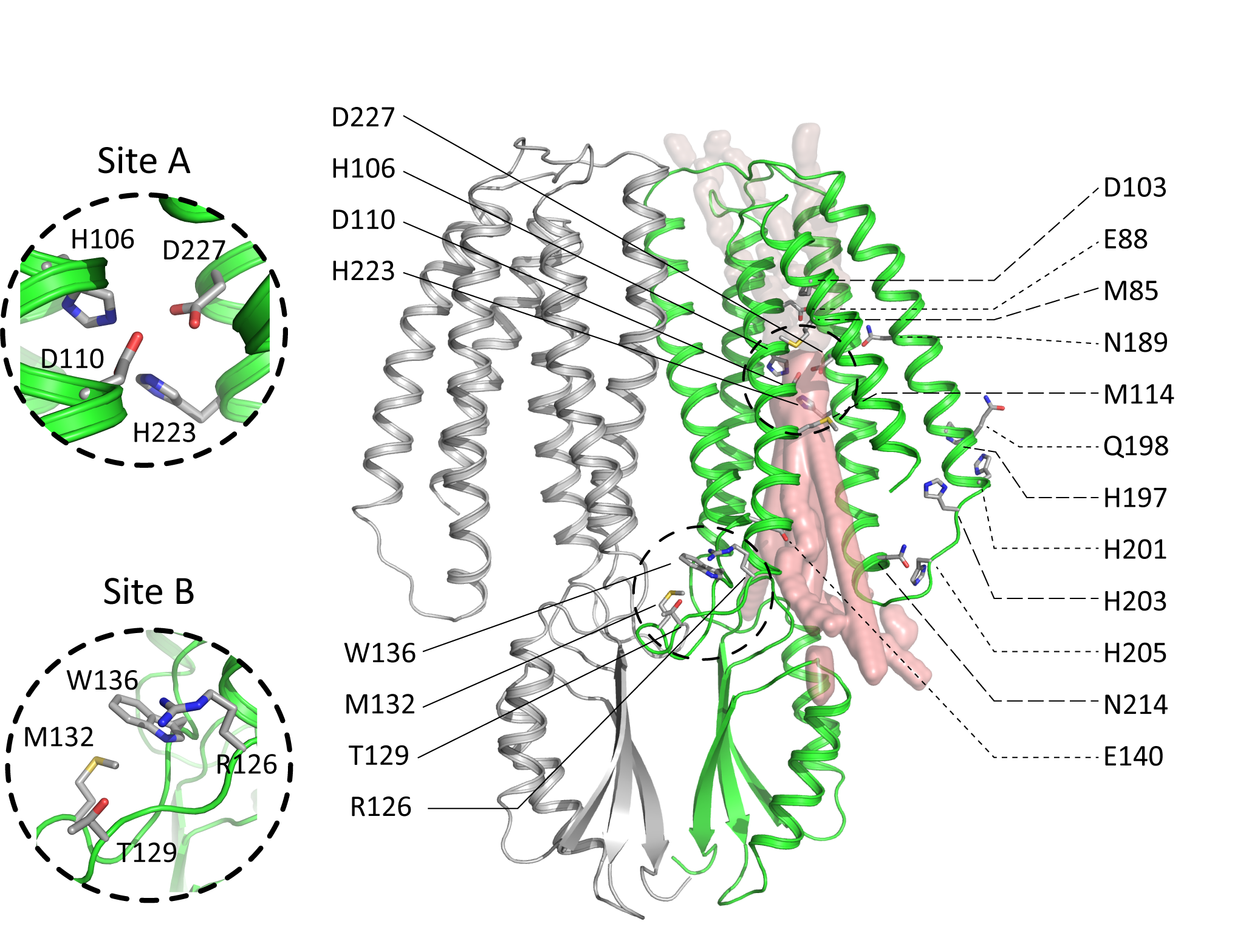

Supplement: S6 Fig — See Fig 3for details. (TIF) [file pcbi.1006503.s008.tif]
